# A Call to Preventive Action by Health Care Providers and Policy Makers to Support Caregivers

**DOI:** 10.5888/pcd13.160233

**Published:** 2016-07-21

**Authors:** David Hoffman, Howard Zucker

**Affiliations:** Author Affiliations: Howard Zucker, New York State Commissioner of Health, Albany, New York. Mr. Hoffman is also Clinical Associate Professor, UAlbany School of Public Health Department of Health Policy Management and Behavior, Albany, New York.

The stress of caring for a vulnerable senior or other person with special needs is a major risk factor for developing numerous chronic conditions. It is also a factor for engaging in risky behaviors such as sedentary lifestyle, poor nutrition, social isolation, and overuse and abuse of substances such as alcohol or prescription drugs. Another common result of the burden of caregiving is absenteeism or presenteeism at work (ie, not going to work at all or going to work while ill) — an additional strain for the individual, family, and employer (1,2).

The health and well-being of our informal caregivers is in many ways the backbone of our system of care — both health care and long-term services and supports rely on the day-to-day care at home to meet the basic needs of the vulnerable elderly or persons with special needs. Those of us in caring professions have both a moral and ethical responsibility to support these hardworking caregivers. Meanwhile, those in policy-making positions have a responsibility to create policies and systems that enable caregivers to perform their duties without compromising their own health and economic well-being (3).

We know that caregivers with knowledge, access to support systems, ability to engage socially, and resources to make healthy choices live healthier and more fulfilling lives. They are also more likely to have the energy to care for their vulnerable loved one, manage other family responsibilities (ie, sandwich generation members — those caring for children and an aging parent), and maintain their occupation (4,5).

One key element to ensuring that a caregiver has the information and access to support is an actively engaged health care provider — either the caregiver’s health care provider or the provider for the person receiving care. We recommend that it be both. There is a collective professional responsibility to tune in to the well-being of people who are caregivers. This tuning in can and should happen both informally (“So how are you doing today?”) and formally (“Let’s check your vital signs while you are here.”). These vital signs should include assessing the caregiver’s sleep, nutrition, physical activity, work/life balance, use of alcohol, and access to support services, including respite.

Gauging caregivers’ knowledge and coping skills can help reduce their burden on multiple fronts. In the case of progressive disease, for instance, we can assess whether the caregiver has a plan for how to manage the increasing physical demands of that role. Similarly, caregiver education on how to communicate with a loved one who has dementia can substantially reduce or prevent dementia-related behaviors, which are linked to a greater impact on caregiver burden than disease stage (6). In addition, knowing that depression and anxiety are often comorbid conditions can increase the caregiver’s ability to manage difficult behaviors effectively, with or without medications. Planning and adapting to new care strategies takes time and may require that health care providers go beyond the clinical setting to link caregivers with community organizations and resources for sustained support.

Finding time for one more activity in a busy medical office, urgent care center, or emergency department is difficult. But waiting until the caregiver has a health crisis does not serve anyone: when caregivers have a health crisis, they may not be able to continue caring for their loved ones. Providers must have easy access to information about caregiver support systems so they can communicate such information to people caring for their patients. We in public health are trying hard to address these information needs with web-based resource directories, professional education opportunities for caregivers, and information about the availability of caregiver support. We will continue to do our part in making information accessible and in fighting for policies that support caregivers, reduce their stress, and enable them to make healthful choices. Policies, such as paid family leave and those that encourage early detection of dementia and advanced preparation of legal documents that put affairs in good order before the onset of illness, can truly make a difference.
